# Breast Implant Illness and Autoimmune/Inflammatory Syndrome Induced by Adjuvants: Mechanistic Rationale and a Practical Multidisciplinary Diagnostic Algorithm

**DOI:** 10.3390/jcm15145721

**Published:** 2026-07-21

**Authors:** Velizar Shivarov, Angel Yordanov, Milena Ivanova, Eva Tsoneva

**Affiliations:** 1 Department of Clinical Hematology, St. Sophia General Hospital, 1618 Sofia, Bulgaria; vshivarov@abv.bg; 2 Department of Experimental Research, Medical University-Pleven, 5800 Pleven, Bulgaria; 3 Department of Gynecologic Oncology, Medical University-Pleven, 5800 Pleven, Bulgaria; 4 Department of Clinical Hematology, Alexandrovska University Hospital, Medical University Sofia, 1431 Sofia, Bulgaria; mivanova@intech.bg; 5 Department of Gynecology, Military Medical Academy, 1330 Sofia, Bulgaria; dretsoneva@gmail.com

**Keywords:** breast implant illness, ASIA syndrome, silicone breast implants, adjuvants, biofilm, inflammasome, autoimmunity, capsulectomy, BIA-ALCL, diagnostic algorithm

## Abstract

Breast implant illness (BII) describes a constellation of systemic symptoms reported by some patients after breast implantation, including fatigue, cognitive dysfunction, arthralgia, myalgia, sicca symptoms, skin manifestations, and mood disturbance. Autoimmune/inflammatory syndrome induced by adjuvants (ASIA) provides a broader conceptual framework in which silicone and other biomaterials may contribute to immune dysregulation in susceptible individuals, although causality remains controversial and no validated diagnostic biomarker exists. This narrative review summarizes current evidence on BII and implant-related ASIA, focusing on epidemiology, clinical presentation, biological plausibility, diagnostic evaluation, explantation outcomes, and implant-associated complications. Proposed mechanisms include chronic foreign-body inflammation, silicone gel bleed and particulate migration, macrophage activation, inflammasome signaling, cytokine dysregulation, bacterial biofilms and biofilm-derived metabolites, adaptive immune activation, autoantibody formation, and host genetic susceptibility. The review also distinguishes BII/ASIA-like presentations from breast implant-associated anaplastic large cell lymphoma and other rare implant-associated malignancies. A practical multidisciplinary diagnostic and management algorithm is proposed to support structured assessment, red-flag triage, exclusion of alternative diagnoses, patient-centered counseling, and individualized decision-making regarding medical management and explantation.

## 1. Introduction

Breast augmentation and implant-based breast reconstruction are widely performed procedures in plastic and reconstructive surgery. Their use spans cosmetic augmentation, post-mastectomy reconstruction, correction of congenital breast asymmetry, and revision surgery. Contemporary implants differ by filler material, shell surface, shape, cohesiveness, and coating, but all currently marketed saline- and silicone-filled devices contain a silicone elastomer shell [1,2]. Patient satisfaction after breast reconstruction or augmentation is generally high [3]; however, breast implants are not lifetime devices and are associated with recognized local complications such as capsular contracture, rupture, malposition, infection, pain, and the need for reoperation [4].

Over the last decade, growing attention has been directed toward systemic symptoms reported by patients with breast implants. The term breast implant illness (BII) is used by both patients and clinicians to describe a constellation of nonspecific systemic complaints, most commonly fatigue, memory or concentration difficulties (“brain fog”), joint and muscle pain, hair loss, weight changes, anxiety, depression, rash, sleep disturbance, and sicca symptoms [5,6]. The U.S. Food and Drug Administration (FDA) has acknowledged reports of these symptoms in women with all types of breast implants, regardless of filling material, shape, or surface characteristics. At the same time, the FDA emphasizes that BII is not currently recognized as a formal medical diagnosis and that no specific diagnostic test or validated criteria are available [5].

Autoimmune/inflammatory syndrome induced by adjuvants (ASIA), introduced by Shoenfeld and Agmon-Levin in 2011 [7], provides a broader conceptual framework for systemic immune phenomena occurring after exposure to adjuvant substances, including silicone, metals, vaccines, medications, infections, and other biomaterials [4]. Within this model, persistent exposure to an adjuvant may activate innate and adaptive immune pathways in genetically or immunologically susceptible individuals. The ASIA framework is clinically appealing because it links exposure, characteristic systemic manifestations, immunological abnormalities, improvement after removal of the inciting stimulus, and potential progression to a defined autoimmune disease. However, the proposed criteria remain imperfectly validated, and their specificity in patients with common nonspecific symptoms continues to be debated [4].

Despite increasing public awareness and growing scientific interest, clinicians continue to face substantial uncertainty regarding diagnosis, causality, and management of patients presenting with systemic symptoms after breast implantation. The central clinical question is therefore not simply whether implant-related systemic illness exists, but how to identify the subgroup of patients in whom implants may plausibly contribute to immune dysregulation; how to distinguish BII- or ASIA-like presentations from unrelated rheumatologic, endocrine, neurologic, infectious, psychiatric, or nociplastic disorders; and how to counsel patients regarding explantation without either dismissing legitimate symptoms or attributing nonspecific complaints to implants prematurely. This review summarizes the current evidence regarding BII and ASIA, reviews the proposed mechanisms of implant-related immune activation, and presents a practical multidisciplinary diagnostic and management algorithm for patients with suspected BII or ASIA.

## 2. Materials and Methods

This narrative review was prepared through a comprehensive evaluation of the published literature on breast implant illness (BII), autoimmune/inflammatory syndrome induced by adjuvants (ASIA), implant-associated complications, and related immunological mechanisms. Relevant peer-reviewed publications, consensus statements, and regulatory documents were reviewed and critically appraised to provide a balanced overview of the current evidence.

AI-assisted figure preparation. The conceptual figures presented in this review were created with the assistance of ChatGPT (OpenAI, San Francisco, CA, USA; GPT-5.5). The AI tool was used only to assist in the initial visual design and organization of the figures based on the authors’ concepts. All scientific content, figure annotations, interpretation, and final design were critically reviewed, revised, and approved by the authors, who take full responsibility for the accuracy and integrity of the figures.

## 3. Breast Implant Types and Evolution of Device Design

Breast implants may be classified according to filler material, shell surface, shape, and gel cohesiveness. The two main filler types are saline-filled and silicone gel-filled implants. Silicone gel implants include highly cohesive devices, often referred to as “gummy bear” implants. Based on shell surface, implants are generally categorized as smooth or textured, while their shape may be round or anatomical. Polyurethane-coated implants represent a less commonly used subgroup designed to reduce capsular contracture and implant displacement, although their use varies across countries and regulatory environments [2].

The evolution of implant design is relevant because shell permeability, gel cohesiveness, and surface architecture can influence local tissue responses and the extent of silicone gel bleed and particulate migration. Early breast augmentation procedures performed in the 1950s and early 1960s used solid alloplastic materials such as polyurethane, polytetrafluoroethylene, and expanded polyvinyl alcohol formaldehyde. These materials were ultimately abandoned because of tissue reactions, firmness, distortion, and patient discomfort. First-generation silicone gel implants, introduced in 1962, featured thick smooth elastomer shells and moderately viscous gel. Second-generation devices, developed during the 1970s, used thinner shells and lower-viscosity gel, which improved softness but also increased gel bleed and microscopic migration of silicone into surrounding periprosthetic tissues [2,8].

Third-generation implants introduced in the 1980s incorporated multilayered elastomer shells and barrier layers to reduce gel bleed, rupture, and extracapsular migration. Following the 1992 FDA restrictions on silicone gel-filled implants, fourth- and fifth-generation devices were produced under stricter regulatory and quality-control standards, with stronger shells, improved gel cohesiveness, additional surface options, and a wider range of shapes [2].

These design modifications have improved mechanical performance and reduced some device-related complications, but they have not eliminated the biological interactions that occur between implants and host tissues.

## 4. Terminology and Clinical Phenotype

Breast implant illness (BII) is best understood as a patient-reported clinical syndrome rather than a single validated disease entity [9,10]. Although BII overlaps conceptually with autoimmune/inflammatory syndrome induced by adjuvants (ASIA), the two terms are not interchangeable. BII is primarily descriptive and implant-specific, whereas ASIA represents a broader mechanistic framework based on immune responses following exposure to adjuvant materials. Some patients with breast implants fulfill proposed ASIA criteria, some develop defined autoimmune diseases, and others experience chronic systemic symptoms without meeting criteria for a recognized autoimmune disorder. This heterogeneity likely contributes to the inconsistent findings observed in epidemiological studies and may explain why strong population-level associations are difficult to demonstrate despite clinically meaningful symptom patterns reported by individual patients [5,6,11,12,13,14].

The clinical presentation is heterogeneous and often nonspecific. Commonly reported symptoms include fatigue, cognitive impairment or “brain fog,” arthralgia, myalgia, morning stiffness, paresthesia, sicca symptoms, rash, alopecia, low-grade fever, sleep disturbance, palpitations, gastrointestinal complaints, anxiety, and depression [10,15]. Symptom onset may occur months or years after implantation and can fluctuate over time, further complicating clinical interpretation.

The U.S. Food and Drug Administration (FDA) reviewed medical device reports submitted between 1 January 2008 and 30 June 2024 and identified 10,318 reports meeting systemic-symptom search criteria after duplicate elimination, with an average time to symptom onset of 5.6 years among reports with sufficient timing data. Frequently reported symptoms included fatigue, joint-related complaints, anxiety, autoimmune disease mentions, brain fog, hair loss, depression, rash, and weight changes [5]. Although such reports are valuable for signal detection, they cannot establish incidence, prevalence, or causality because passive surveillance systems are inherently limited by under-reporting, duplicate reporting, reporting bias, incomplete clinical verification, and the absence of denominator data [5].

Several observational studies have reported symptom improvement after explantation in a substantial proportion of patients, particularly with respect to fatigue, joint pain, and muscle pain [6]. However, outcomes remain variable, follow-up periods differ considerably across studies, and much of the available literature relies on self-reported outcomes. From a clinical perspective, these findings suggest that explantation may be a reasonable option for selected patients following structured evaluation and shared decision-making, but it should not be presented as a universally effective or curative intervention.

## 5. Mechanistic Rationale for Implant-Related Systemic Symptoms

**Figure 1:**
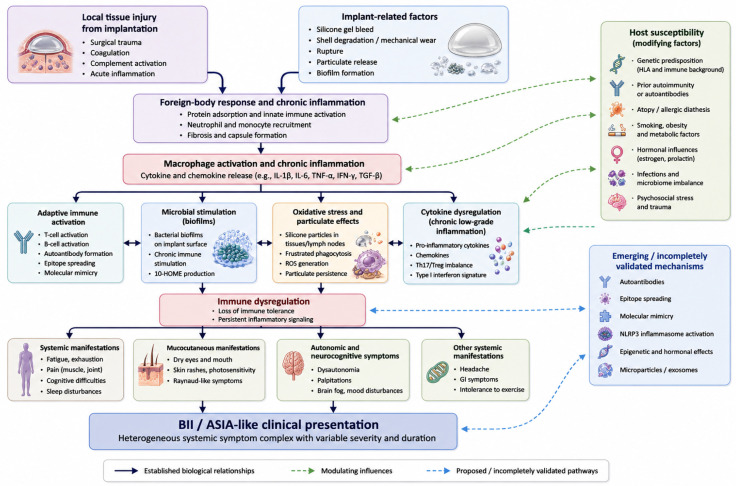
Proposed immunological mechanisms underlying breast implant illness (BII)/ASIA syndrome.

The available mechanistic evidence does not support a single linear pathway linking breast implantation to systemic disease. Rather, BII- and ASIA-like presentations are more likely to arise from the interaction of multiple factors, including implant characteristics, microbial influences, tissue injury, and individual host susceptibility. In this model, chronic immune activation develops when these factors converge in a susceptible individual and exceed a threshold for persistent inflammatory signaling. The mechanistic domains discussed below are not mutually exclusive and may act in parallel or sequentially. A schematic overview of the biologically plausible pathways proposed in the literature is presented in Figure 1.

All breast implants induce a foreign-body response. Following implantation, tissue injury triggers coagulation, complement activation, and an acute inflammatory reaction [16]. Proteins rapidly adsorb onto the implant surface, initiating recruitment of neutrophils and monocytes, followed by macrophage adhesion, foreign-body giant-cell formation, fibroblast activation, collagen deposition, and ultimately capsule formation [16]. In most patients, this response stabilizes and becomes clinically silent [16,17]. In others, however, persistent inflammation and fibrosis may contribute to pain, capsular contracture, implant distortion, and potentially low-grade systemic inflammatory signaling.

Silicone has traditionally been regarded as chemically inert; however, chemical inertness does not necessarily imply immunological invisibility. Silicone gel bleed, shell degradation, rupture, and mechanical wear may expose macrophages and dendritic cells to silicone particles. Silicone-containing vacuoles and particulate material have been identified in periprosthetic tissues and regional lymph nodes [8,16]. Once phagocytosed, these particles may promote chronic macrophage activation, frustrated phagocytosis, oxidative stress, and cytokine release. Although silicone itself is not considered antigenic, its persistent presence may act as an adjuvant, enhancing immune responses to endogenous or exogenous antigens in susceptible individuals [4,16,18]. While these observations support the biological plausibility of silicone-related immune activation, the extent to which silicone particle migration contributes directly to systemic symptoms remains uncertain. Most available evidence derives from pathological, experimental, and translational studies rather than prospective clinical investigations.

### 5.1. Innate Immune Activation, Inflammasome Signaling, and Cytokine Amplification

Innate immune activation has been proposed as an important component of the biological mechanisms underlying implant-related systemic symptoms. Macrophages exposed to particulate material, damage-associated signals, bacterial products, or oxidized lipids may activate pattern-recognition pathways, resulting in the release of proinflammatory cytokines, chemokines, and profibrotic mediators [8,14,16,19]. Among the proposed mechanisms, activation of the NLRP3 inflammasome has attracted particular interest because it provides a biologically plausible link between particulate or danger-associated signals and the production of interleukin-1β and interleukin-18 [20,21]. However, these pathways remain primarily mechanistic, and routine clinical assessment of inflammasome activity is not currently validated for diagnostic purposes. Although inflammasome activation represents a compelling mechanistic hypothesis, evidence linking these pathways directly to the clinical manifestations of BII remains limited. Further studies are required to determine whether inflammasome-related biomarkers possess diagnostic or prognostic utility in patients with suspected BII or ASIA.

Persistent low-grade cytokine signaling may contribute to constitutional symptoms such as fatigue, sleep disturbance, cognitive dysfunction, myalgia, and mood changes [22]. At the same time, these symptoms are not specific to BII and represent common downstream manifestations of a wide range of inflammatory, endocrine, infectious, psychiatric, and nociplastic disorders. Their presence should therefore prompt a structured differential diagnostic evaluation rather than immediate attribution to breast implants.

### 5.2. Biofilms and Host–Microbe Immune Interactions

Biofilm formation may represent an important link between a local implant environment and systemic immune manifestations. Small bacterial communities can persist on implant surfaces, particularly when embedded within an extracellular polymeric matrix that protects them from host defenses and conventional culture techniques. As a result, biofilms may provide a source of chronic or intermittent immune stimulation without causing overt clinical infection. Organisms most commonly discussed in the breast implant literature include Staphylococcus epidermidis, Staphylococcus aureus, Cutibacterium acnes, and other skin commensals, although the true biofilm burden may be underestimated by standard culture-based methods [23,24].

Recent studies have drawn attention to biofilm-derived metabolites as potential mediators of implant-associated inflammation. In an analysis of explanted breast implants, increased biofilm burden was associated with the oxylipin 10-HOME on implant surfaces. Experimental exposure to 10-HOME promoted Th1-skewed immune responses and proinflammatory macrophage differentiation in model systems [25]. Although these findings do not establish biofilms as the sole explanation for BII, they provide a potential molecular link between implant-associated microorganisms, lipid metabolites, macrophage polarization, and systemic immune responses. Despite increasing interest in biofilm-associated mechanisms, their precise contribution to systemic symptoms remains unclear. Current evidence supports a potential role for chronic microbial stimulation in selected patients, but larger prospective studies are needed to establish clinical relevance and causality.

### 5.3. Surface Topography, Textured Implants, and Chronic Inflammatory Load

Implant surface characteristics influence both host tissue responses and microbial adhesion. Compared with smooth implants, textured and macrotextured surfaces provide a larger surface area and may promote greater mechanical friction, cellular entrapment, and bacterial adherence. Experimental and translational studies suggest that surface roughness can affect local cytokine profiles, leukocyte behavior, macrophage activation, and fibrotic responses, highlighting the role of implant topography in shaping the periprosthetic inflammatory environment [26,27].

The clinical relevance of implant surface characteristics is perhaps best illustrated by breast implant-associated anaplastic large cell lymphoma (BIA-ALCL). The association between textured implants and BIA-ALCL is substantially better established than any association between implant surface type and BII. BIA-ALCL is a distinct CD30-positive, usually ALK-negative T-cell lymphoma that typically presents years after implantation with delayed seroma, breast swelling, pain, or a capsular mass [22,28]. Although its pathogenesis is not fully understood, current evidence suggests that chronic antigenic stimulation, biofilm-associated inflammation, implant surface characteristics, and host susceptibility may all contribute to disease development.

Importantly, BIA-ALCL and BII are distinct clinical entities and should not be conflated. Nevertheless, the existence of BIA-ALCL demonstrates that chronic implant-associated inflammation can, in rare circumstances, have clinically significant immunological consequences. This observation does not establish a causal relationship between implant surface characteristics and BII, but it supports the broader concept that long-term interactions between implant materials, the local microenvironment, and the host immune system may influence clinical outcomes. Notably, evidence linking implant surface characteristics to BII remains considerably weaker than that linking textured implants to BIA-ALCL. Although implant topography may influence the local inflammatory environment, its role in systemic symptom development has yet to be clearly defined.

Although textured implants have been more consistently associated with chronic local inflammation and the development of BIA-ALCL, systemic symptoms compatible with breast implant illness have been described in patients with both smooth and textured implants. In a prospective cohort study of women undergoing explantation for presumed BII, Wee et al. found no significant association between implant surface characteristics and symptom improvement after implant removal, suggesting that BII cannot be explained solely by implant surface type [29]. On the other hand, experimental data indicate that surface topography may influence the local immune response. Vinci et al. demonstrated that macrotextured implant surfaces induced greater leukocyte activation and increased expression of pro-inflammatory cytokines compared with microtextured surfaces, supporting the concept that implant surface characteristics may contribute to a more pronounced chronic inflammatory microenvironment [26]. However, these findings primarily relate to local tissue responses and should not be interpreted as evidence that textured implants independently increase the risk of systemic disease. The role of implant geometry remains even less clear. Although both round and anatomical implants are widely used in clinical practice, the available literature provides little evidence that implant shape itself influences the development of BII or ASIA. Because anatomical implants have historically been manufactured predominantly with textured surfaces to reduce implant rotation, the potential effects of implant shape and surface characteristics are difficult to separate. Further prospective studies specifically evaluating implant design are therefore needed before any conclusions can be drawn regarding the independent contribution of implant shape to systemic symptoms.

### 5.4. Adaptive Autoimmunity, Autoantibodies, and Genetic Susceptibility

Persistent activation of innate immune pathways may, in some individuals, contribute to the development of adaptive immune responses and autoimmune phenomena. Several mechanisms have been proposed, including enhanced antigen presentation, bystander activation, epitope spreading following tissue injury, molecular mimicry involving microbial antigens, B-cell activation, and loss of peripheral tolerance. While none of these mechanisms has been conclusively demonstrated in implant-related disease, together they provide a biologically plausible framework linking chronic inflammation to the emergence of autoimmunity.

Autoantibodies, including antinuclear antibodies, anti-thyroid antibodies, rheumatoid factor, anti-cyclic citrullinated peptide antibodies, and extractable nuclear antigen antibodies, have been reported in some patients with breast implants [4,14,16]. However, these findings are not specific and should be interpreted within the broader clinical context rather than as evidence of implant-related disease.

Host genetic factors may also influence susceptibility. Associations involving HLA-DRB1 and HLA-DQ have been proposed [30] and are biologically plausible given the central role of HLA class II molecules in antigen presentation, CD4+ T-cell responses, and immune tolerance. However, the available evidence remains limited and is insufficient to support clinical risk stratification or predictive testing at present [4,14,16]. Currently, no autoantibody profile or genetic marker has demonstrated sufficient specificity or predictive value to support routine clinical use. Reported associations should therefore be considered hypothesis-generating rather than diagnostic.

Future research will likely require integrated prospective studies that combine genetic profiling, immune phenotyping, microbiome analysis, implant characteristics, and long-term clinical outcomes. Such approaches may help clarify why only a subset of patients develop systemic symptoms despite widespread exposure to similar implant materials.

### 5.5. Neuroimmune and Nociplastic Overlap

A comprehensive model must also recognize neuroimmune and nociplastic mechanisms. Chronic inflammation, sleep disruption, pain, anxiety, and diagnostic uncertainty may reinforce one another through central sensitization, autonomic dysregulation, and altered stress-response pathways [22]. This does not imply that symptoms are “psychogenic.” Rather, it acknowledges that persistent immune and tissue signals can become amplified through nervous system mechanisms. Evaluation should therefore include mental health and sleep assessment when clinically relevant, while avoiding dismissal of symptoms as purely psychiatric (Table 1).

Taken together, the available evidence suggests that implant-related systemic symptoms are unlikely to be explained by a single pathogenic mechanism. Rather, foreign-body inflammation, silicone exposure, microbial factors, adaptive immune responses, and host susceptibility may interact in complex and overlapping ways. Some of these processes, such as capsule formation and chronic local inflammation, are well-established biological responses to implanted materials [16,17,19,34,36], whereas others remain emerging hypotheses requiring further validation. At present, no single mechanism adequately accounts for the full spectrum of reported symptoms, underscoring the need for continued multidisciplinary research.

## 6. Diagnostic Approach: Principles Before Algorithm

**Figure 2:**
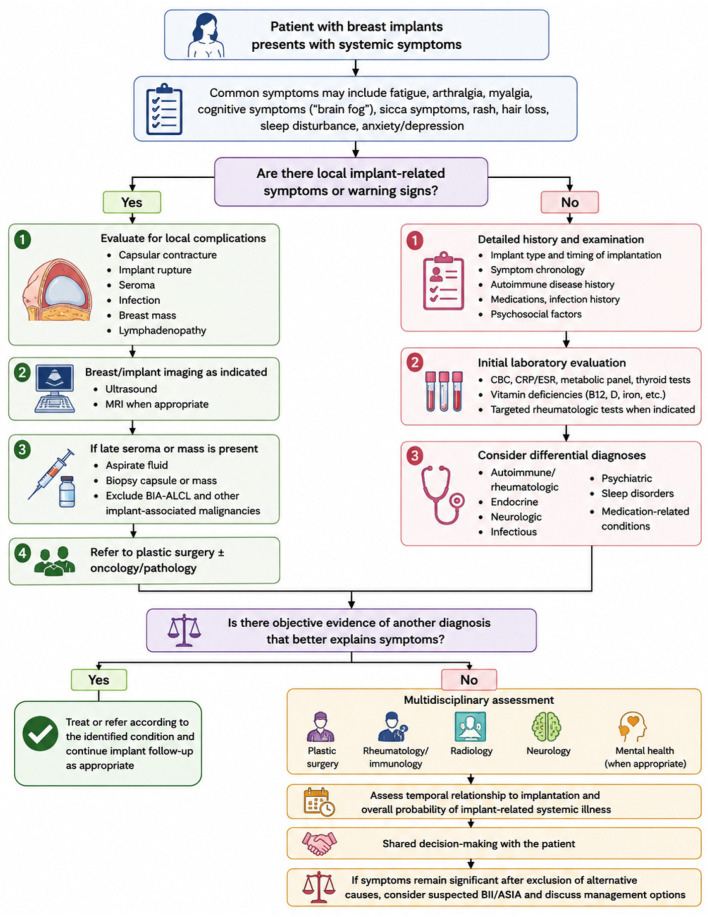
Proposed diagnostic algorithm for suspected breast implant illness (BII)/ASIA syndrome.

There is no validated laboratory test for BII and no universally accepted classification system for implant-related ASIA [4,5,11,18]. Diagnosis is therefore clinical and probabilistic. The diagnostic objective is not to “prove” implant causality at first visit; it is to identify urgent implant-related complications, exclude common alternative diagnoses, phenotype symptoms, document temporal relationships, assess objective immune or implant abnormalities, and guide shared decision-making. A practical overview of the proposed diagnostic pathway is presented in Figure 2.

The diagnostic and management algorithm was developed by synthesizing the evidence reviewed for this article, including published guidelines, consensus recommendations, regulatory documents, and clinical studies addressing BII, ASIA, implant-related complications, and explantation outcomes. Where the available evidence was limited or inconsistent, the proposed steps reflect the authors’ clinical interpretation of the literature. The algorithm is intended as a practical framework to support multidisciplinary assessment and shared decision-making, rather than as a formally validated clinical guideline.

The proposed pathway separates two tracks. The first is an urgent red-flag track for possible implant-associated malignancy or significant local implant complication. The second is a systemic-symptom track for suspected BII/ASIA. This separation is essential because BIA-ALCL, BIA-SCC, infection, rupture, and capsular masses require different investigations and different surgical/pathological standards than nonspecific systemic symptoms [22,24,28].

### 6.1. Red Flags Requiring Urgent Implant-Focused Evaluation

Delayed peri-implant seroma, new unilateral breast swelling, breast asymmetry, persistent breast pain, palpable capsular or breast mass, regional lymphadenopathy, skin ulceration or discoloration, recurrent peri-implant inflammation, constitutional “B symptoms,” or rapidly progressive local findings.

For late seroma or mass, initial evaluation should include breast/implant ultrasound and aspiration of fluid when present. Seroma fluid should be sent for cytology, CD30 immunohistochemistry, ALK status, flow cytometry when appropriate, and microbiology according to local protocols. Suspicious masses or abnormal nodes require biopsy and multidisciplinary referral. MRI, CT, or PET-CT should be selected according to the suspected diagnosis and local oncologic guidance [22,23,24,28]. The proposed multidisciplinary diagnostic and management algorithm is summarized in Table 2.

### 6.2. Working Diagnostic Categories

Because BII lacks validated diagnostic criteria, clinical communication benefits from a transparent probability language [4,5,8,14,18,41]. “Probable implant-related systemic illness” may be used when there is a typical multisystem symptom cluster, a plausible temporal relationship to implantation or revision, absence of a better diagnosis after appropriate evaluation, and at least one supportive feature such as implant rupture, capsular inflammation, biofilm/infection evidence, autoantibody or inflammatory abnormalities, fulfillment of proposed ASIA criteria [4,11,18], or sustained improvement after explantation [6,21,42]. “Possible implant-related systemic illness” describes incomplete or mixed cases in which symptoms are compatible but temporality, workup, or objective support is limited. “Unlikely implant-related systemic illness” should be used when another diagnosis more convincingly explains the presentation or when the symptom pattern has no clinical or temporal coherence with implant exposure.

These categories are not intended to replace formal rheumatologic, neurologic, psychiatric, oncologic, or plastic-surgical diagnoses. They are pragmatic labels for multidisciplinary decision-making, patient counseling, and longitudinal follow-up.

### 6.3. Common Diagnostic Pitfalls

Several recurring pitfalls may complicate the evaluation of patients with suspected BII. These include assuming implant causality at the initial consultation, attributing all symptoms to breast implants without adequate investigation, overinterpreting nonspecific autoantibody findings, and presenting explantation as a guaranteed treatment. Equally problematic is the dismissal of patient-reported symptoms without appropriate evaluation. A balanced approach requires careful assessment of alternative diagnoses while remaining open to the possibility that implant-related factors may contribute to symptoms in selected individuals [6,8,14]. A balanced approach requires careful assessment of alternative diagnoses while remaining open to the possibility that implant-related factors may contribute to symptoms in selected individuals. Practical communication strategies to support this patient-centered approach are discussed in the following section.

## 7. Management

### 7.1. Patient-Centered Counseling

Patients presenting with suspected BII or ASIA often describe prolonged diagnostic uncertainty and perceived dismissal. The first therapeutic intervention is therefore a credible, structured consultation. Clinicians should acknowledge symptoms as real, explain the uncertainty of causality, distinguish BII from BIA-ALCL and other capsule-associated malignancies, and outline a staged evaluation. The aim is neither to validate every symptom as implant-caused nor to deny a possible implant contribution before evaluation.

Counseling should include the following points: BII is not currently a formal diagnosis with validated criteria; symptoms have been reported with saline and silicone implants as well as smooth and textured devices; some patients report improvement after explantation, but resolution is not universal; and explantation does not eliminate the need to diagnose and treat coexisting autoimmune, endocrine, neurologic, psychiatric, or pain conditions [5,6,21,42].

### 7.2. Medical Management

When evaluation identifies a defined autoimmune disease, management should follow disease-specific standards. Sjögren syndrome, rheumatoid arthritis, systemic sclerosis, autoimmune thyroid disease, inflammatory myopathy, vasculitis, and connective tissue disease should not be reduced to “implant illness” alone. Immunomodulatory therapy may be appropriate when objective inflammatory disease is present [4,8,14]. Conversely, prolonged empirical corticosteroid or immunosuppressive therapy for nonspecific symptoms without objective inflammation should be avoided unless justified by a specialist assessment.

Symptom-directed care remains important. Sleep optimization, graded physical rehabilitation, pain-modulating strategies, treatment of mood disorders, management of sicca symptoms, and correction of metabolic deficiencies may improve quality of life regardless of the final attribution. This supportive care should be offered without implying that symptoms are imagined.

### 7.3. Explantation and Capsulectomy

Explantation is the most discussed intervention for BII/ASIA-like presentations. Observational studies and prospective cohorts show that many patients report partial or complete symptom improvement after implant removal, but the magnitude and durability of benefit vary [5,6,21,42]. The decision should be individualized and based on symptom burden, temporal association, exclusion of alternative diagnoses, implant integrity, local complications, patient values, surgical risk, and realistic expectations. A corresponding management pathway, including observation, supportive care, and shared decision-making regarding explantation, is shown in Figure 2.

The extent of capsulectomy should be determined by clinical indication and surgical safety. Total capsulectomy may be appropriate for capsular contracture, rupture with silicone contamination, recurrent seroma, abnormal capsule, or patient preference after informed discussion. However, current consensus terminology reserves “en bloc capsulectomy” for removal of the implant capsule with a margin of uninvolved tissue in cases of suspected or established implant-associated cancer after appropriate workup [36]. Routine use of the term “en bloc” for BII alone may confuse patients and should be avoided.

In patients with suspected BII without evidence of implant-associated malignancy, the extent of capsulectomy should be individualized based on the clinical findings, implant status, and surgical risk. Partial capsulectomy may be appropriate when complete capsule removal is not technically feasible or would increase operative risk, whereas total capsulectomy may be considered in the presence of significant capsular pathology, implant rupture with silicone leakage, or severe capsular contracture. Patients should also be informed that current evidence does not demonstrate that more extensive capsulectomy consistently results in greater improvement of systemic symptoms [39].

When explantation is performed for systemic symptoms, operative documentation should include implant type, capsule appearance, rupture status, silicone leakage, seroma, masses, calcification, and whether partial, total, total intact, or en bloc capsulectomy was performed. Tissue should be submitted for histopathology when the capsule is abnormal, when there is fluid or mass, when the implant is textured and late seroma is present, or when local symptoms are unexplained. Microbiological culture or molecular biofilm analysis may be considered when infection or biofilm-related inflammation is suspected, recognizing that routine methods are imperfect [23,24,25].

### 7.4. BIA-ALCL, BIA-SCC and Other Implant-Associated Malignancies: Separation from BII/ASIA

BIA-ALCL is distinct from BII and ASIA. It is an uncommon T-cell lymphoma, most frequently associated with textured implants, typically presenting with delayed peri-implant seroma, swelling, pain, capsular mass, or lymphadenopathy years after implantation [22,28]. The FDA reported 1380 medical device reports of BIA-ALCL and 64 deaths in its MDR review through 30 June 2024, while emphasizing the limitations of passive surveillance data [22]. Any suspected case requires implant-focused imaging, fluid aspiration or tissue biopsy, cytology, CD30 testing, ALK assessment, and multidisciplinary management.

The FDA has also issued safety communications regarding rare cases of squamous cell carcinoma and other lymphomas arising in the capsule around breast implants, separate from BIA-ALCL. These events appear rare, and the FDA does not recommend removal of asymptomatic implants solely because of concern about these malignancies. However, new swelling, pain, mass, or skin changes should prompt medical evaluation [24]. Including these entities in the algorithm reduces the risk of falsely reassuring patients with local red flags under the umbrella of BII.

## 8. Current Controversies and Limitations of the Evidence

Despite growing awareness of BII and increasing interest in implant-related immune phenomena, important questions remain unanswered. Much of the ongoing debate reflects the gap between mechanistic plausibility and clinical proof. On the one hand, a growing body of experimental and translational research supports several pathways through which breast implants could contribute to chronic immune activation in susceptible individuals. On the other hand, epidemiological studies have produced inconsistent results, and a definitive causal relationship between breast implants and systemic disease has not been established [6,8,12,13,14].

A major challenge is that BII remains a descriptive clinical term rather than a formally defined disease entity. The symptoms most frequently attributed to BII—including fatigue, cognitive dysfunction, musculoskeletal pain, sleep disturbance, and mood changes—are common in the general population and overlap with a wide range of autoimmune, endocrine, neurologic, infectious, and psychiatric conditions. In the absence of validated diagnostic criteria or disease-specific biomarkers, determining whether breast implants contribute to an individual patient’s symptoms remains inherently difficult [5,6,8,14].

Similar uncertainties apply to the ASIA framework. Although ASIA provides a useful conceptual model for understanding immune responses to adjuvant materials, its diagnostic criteria remain incompletely validated and their specificity continues to be debated. In addition, the sensitivity and specificity of the proposed ASIA criteria have not been established in prospective validation studies, limiting their use as a diagnostic tool in routine clinical practice. Supporters view ASIA as a clinically valuable framework that helps connect exposure history, immunological abnormalities, and symptom patterns, whereas critics argue that its broad criteria may increase the risk of attributing nonspecific symptoms to adjuvant exposure without sufficient objective evidence [4,16,18].

Another area of uncertainty concerns the apparent discrepancy between epidemiological findings and mechanistic studies. Large population-based investigations and meta-analyses have generally failed to demonstrate a substantial increase in classical autoimmune diseases among women with breast implants. At the same time, studies of capsule biology, silicone particle migration, biofilms, inflammasome activation, and host susceptibility continue to identify mechanisms that could plausibly contribute to immune dysregulation in selected individuals. These observations are not necessarily contradictory and may simply reflect the heterogeneity of the patient population, in which only a subset of exposed individuals develop clinically significant symptoms [8,13,14,25].

Interpretation of explantation studies also requires caution. Many patients report symptomatic improvement after implant removal, and these observations should not be dismissed. However, reported outcomes vary considerably between studies, follow-up periods are often limited, and many investigations rely heavily on patient-reported measures. Improvement after explantation may support a potential implant contribution, but it does not by itself establish causality [6,21,42].

Taken together, the available evidence supports neither outright dismissal nor unquestioning acceptance of implant-related systemic illness. Instead, it points toward a complex and evolving field in which biological mechanisms, individual susceptibility, and clinical presentation may intersect in ways that are not yet fully understood [6,8,14]. Future progress will depend on well-designed prospective studies incorporating standardized symptom assessment, implant characteristics, imaging findings, pathology, microbiological analysis, immunological profiling, and long-term clinical outcomes.

## 9. Future Directions

Despite the growing body of literature on BII and implant-related ASIA, many important questions remain unanswered. One of the greatest challenges is the absence of validated diagnostic criteria and objective biomarkers, making it difficult to distinguish implant-related illness from other conditions that present with similar symptoms [4,5,6,8,14]. As a result, much of the current evidence relies on patient-reported outcomes, observational studies, and retrospective analyses, all of which have inherent limitations [6,8,12,13,14].

Future research should focus on prospective studies that follow patients over time and use standardized methods for symptom assessment and outcome reporting. A better understanding of the relationship between clinical symptoms, implant characteristics, imaging findings, capsule pathology, microbiological factors, and immunological changes may help identify patient subgroups that are particularly susceptible to implant-related immune responses.

National breast implant registries and post-market surveillance programs are also likely to contribute to a better understanding of BII in the coming years. By systematically collecting information on implant characteristics, surgical procedures, long-term outcomes, and patient-reported symptoms, these registries may help identify potential risk factors, detect safety signals earlier, and provide more robust real-world evidence than is currently available. Continued international collaboration and harmonization of registry data may further improve our understanding of the incidence, natural history, and clinical spectrum of BII [43,44].

Another important area of investigation is individual susceptibility. The fact that many women with breast implants remain asymptomatic while others develop systemic symptoms suggests that implant exposure alone is unlikely to explain the full clinical picture [8,13,14]. Genetic predisposition, immune regulation, microbial factors, hormonal influences, and environmental exposures may all contribute to differences in clinical presentation and deserve further study.

Additional research is also needed to clarify the role of explantation. Although many patients report symptomatic improvement after implant removal, outcomes are variable and predictors of response remain poorly defined [6,21,42]. Identifying which patients are most likely to benefit from surgical intervention would improve counseling, facilitate shared decision-making, and help set realistic expectations.

Several specific research priorities should also be addressed. Future studies should determine whether distinct genetic, immunological, or microbiological profiles can identify patients who are particularly susceptible to implant-related systemic symptoms. Additional research is needed to clarify whether implant characteristics, such as surface type, shell design, or gel cohesiveness, independently influence the risk of BII. The identification of reliable diagnostic and prognostic biomarkers remains another major priority, as does the development of prospective studies to determine which patients are most likely to benefit from explantation and whether the extent of capsulectomy influences long-term clinical outcomes.

Ultimately, progress in this field will require close collaboration between plastic surgeons, rheumatologists, immunologists, microbiologists, pathologists, and epidemiologists. Such multidisciplinary efforts are likely to provide a more complete understanding of implant-related systemic symptoms and may eventually lead to more precise diagnostic tools and individualized treatment strategies.

## 10. Discussion

The literature on BII and ASIA is characterized by a tension between epidemiologic uncertainty and mechanistic plausibility. Large meta-analyses and systematic reviews of connective tissue disease have not consistently shown major increases in classical autoimmune diseases among women with implants [12,13,14]. At the same time, patient-reported systemic symptoms are repeatedly documented, passive surveillance systems capture thousands of reports, explantation cohorts frequently show improvement in selected patients, and mechanistic studies increasingly identify plausible pathways involving foreign-body inflammation, silicone particles, microbial biofilms, oxylipins, surface topography, and host immune susceptibility [5,6,21,25,26,42].

These observations can be reconciled if implant-related systemic illness is viewed as a heterogeneous syndrome rather than a single disease. A small susceptible sub-group may develop defined autoimmune disease; another subgroup may develop chronic inflammatory or neuroimmune symptoms without meeting autoimmune criteria; and a third group may have symptoms driven primarily by unrelated conditions. The clinical task is to distinguish these groups as accurately as possible.

The algorithm proposed here is intentionally conservative. It avoids both extremes: automatic dismissal of symptoms because BII lacks formal criteria [9], and automatic recommendation of explantation without a medical workup. It also separates systemic-symptom evaluation from oncologic red flags, which is essential because BIA-ALCL and BIA-SCC require urgent diagnostic pathways distinct from BII/ASIA counseling [45].

Future studies should prioritize prospective longitudinal cohorts with pre-implant baseline data, standardized symptom instruments, device registries, HLA and immunogenetic profiling, high-quality implant imaging, capsule histopathology, bio-film/metabolomic analysis, and long-term outcomes after different surgical strategies. Only such integrated datasets can determine which biomarkers predict disease, which patients benefit most from explantation, and which capsulectomy approaches are necessary for specific clinical scenarios.

Until such evidence becomes available, clinicians should adopt a balanced, multi-disciplinary approach that neither dismisses patient-reported symptoms nor assumes implant causality in the absence of appropriate evaluation.

## 11. Conclusions

Breast implant illness and implant-related ASIA remain challenging and often controversial topics at the intersection of plastic surgery, immunology, rheumatology, and patient-centered medicine. While a definitive causal relationship between breast implants and systemic disease has not been established [14], accumulating mechanistic and clinical evidence suggests that implant-related immune dysregulation may occur in a subset of susceptible individuals [46]. At the same time, the heterogeneity of reported symptoms, the absence of validated biomarkers, and the limitations of existing epidemiological studies continue to complicate diagnosis and clinical decision-making.

The challenge for clinicians is not simply determining whether breast implants can contribute to systemic symptoms, but identifying the patients in whom such a contribution is plausible while carefully excluding alternative explanations. This requires a structured and multidisciplinary approach that combines symptom phenotyping, assessment of implant integrity and local complications, targeted laboratory evaluation, and thoughtful consideration of individual risk factors.

Patients presenting with systemic symptoms deserve to be heard and evaluated without prejudice. However, neither implant causality nor symptom dismissal should be the default position. A balanced, evidence-based approach remains essential, particularly when discussing explantation and other potentially irreversible interventions.

Future progress will depend on well-designed prospective studies integrating clinical outcomes, implant characteristics, immunological profiling, genetics, microbiome data, and capsule pathology. Such work may help clarify the biological mechanisms involved, identify patients at greatest risk, and ultimately move the field beyond debate toward more precise diagnosis and individualized care.

## Figures and Tables

**Table 1:** Mechanistic domains linking breast implants to systemic symptoms.

Mechanistic Domain	Principal Triggers	Immune/Tissue Pathway	Possible Clinical Significance
Foreign-body response [20]	Implant surface, tissue injury, chronic capsule formation [20]	Macrophages, foreign-body giant cells, fibroblasts, collagen deposition, cytokines [20]	May explain pain, contracture, local inflammation and low-grade systemic signaling [20]
Silicone exposure [31]	Gel bleed, shell wear, rupture, particulate migration [31]	Particle uptake, frustrated phagocytosis, oxidative stress, macrophage activation [32]	Supports adjuvant plausibility but is not diagnostic by itself [33]
Inflammasome signaling [20]	Particulates, danger signals, bacterial products [34]	NLRP3-type pathways, IL-1β/IL-18 release, downstream IL-6/TNF-α [35]	Plausible contributor to fatigue, myalgia, feverishness and inflammatory symptoms [34]
Biofilm [31]	Low-grade microbial colonization, especially skin commensals	Persistent innate stimulation; biofilm-derived metabolites such as 10-HOME; Th1 skewing [36]	Consider in capsular contracture, recurrent inflammation, seroma, or explant specimens [37]
Surface topography [31]	Textured or macrotextured surfaces, friction, increased area [31]	Leukocyte entrapment, cytokine shifts, biofilm adherence, chronic inflammatory microenvironment [19]	Important for BIA-ALCL risk assessment and possibly inflammatory burden [31]
Adaptive autoimmunity [33]	Persistent antigen presentation, epitope spreading, molecular mimicry [33]	Autoreactive T/B cells, autoantibodies, evolution toward defined autoimmune disease [33]	Requires standard rheumatologic workup; autoantibodies are supportive but nonspecific [32]
Host susceptibility [38]	HLA class II background, prior autoimmunity, atopy, infection, smoking, obesity, psychosocial stressors [38]	Lower threshold for immune activation or symptom amplification [38]	May explain why only a subset of patients develop systemic symptoms [38]

**Table 2:** Proposed multidisciplinary diagnostic and management algorithm for suspected BII/ASIA.

Step	Clinical Action	Minimum Content	Interpretation/Next Decision
1. Confirm implant history	Collect device and surgical data	Date and indication; implant fill, surface, shape, brand/model if available; plane; revisions; rupture history; capsular contracture; textured-device exposure; radiotherapy; infections; pregnancy/postpartum timing.	Defines exposure, latency and implant-specific risk profile. Missing device data should not stop evaluation, but should be documented.
2. Triage red flags	Separate malignant/local-complication pathway from systemic-symptom pathway	Delayed seroma, swelling, mass, lymphadenopathy, skin changes, persistent focal pain, recurrent inflammation, B symptoms.	If present, prioritize imaging, aspiration/biopsy, CD30/ALK testing, pathology, and multidisciplinary referral before attributing symptoms to BII.
3. Phenotype symptoms	Map symptoms by domain and chronology	Constitutional, musculoskeletal, sicca, dermatologic, neurologic, cognitive, autonomic, endocrine, psychiatric, sleep, gastrointestinal; onset relative to implantation/revision/rupture; fluctuation; triggers.	A temporally coherent multisystem cluster increases plausibility; isolated nonspecific symptoms require broad differential diagnosis.
4. Exclude common alternative diagnoses	Perform structured medical assessment	Medication review; pregnancy/menopause; thyroid disease; anemia; diabetes; vitamin deficiency; chronic infection; malignancy; inflammatory rheumatic disease; fibromyalgia/nociplastic pain; sleep apnea; depression/anxiety; occupational exposures.	A positive alternative diagnosis should be treated. Implant contribution may still be possible, but should not replace standard care.
5. Baseline laboratory screen	Order first-line tests and targeted immunology	CBC with differential, ESR/CRP, CMP, urinalysis/proteinuria, TSH/free T4, ferritin, B12/folate/vitamin D, CK if myalgia/weakness, ANA with reflex ENA/dsDNA/complement, RF/anti-CCP, thyroid antibodies, immunoglobulins when infections or immune dysregulation are suspected.	Normal tests do not exclude BII [39]. Abnormal tests should guide referral to rheumatology, immunology, endocrinology, neurology, infectious diseases, or hematology as appropriate.
6. Assess implant integrity and capsule	Use imaging according to symptoms and device type	Ultrasound for seroma, mass, capsular abnormality or rupture screening; MRI for suspected silicone rupture or equivocal ultrasound; mammography/oncologic imaging according to age and cancer-screening indication.	Objective rupture, seroma, mass, capsular contracture or inflammatory findings increase the rationale for plastic-surgery evaluation and possible explantation.
7. Apply working probability category	Classify as probable, possible, or unlikely implant-related systemic illness	Probable: typical multisystem symptoms, plausible latency, no better explanation after workup, supportive immune/implant abnormality or improvement after explantation. Possible: partial temporal relation or incomplete workup. Unlikely: strong alternative diagnosis or no temporal/clinical coherence.	This is a clinical working classification, not a validated diagnosis. It should be updated after follow-up, test results and treatment response.
8. Shared management plan	Choose conservative, medical, surgical, or combined pathway	Treat defined autoimmune/endocrine/infectious disease; symptom-directed care; mental health/sleep/pain support; discuss risks and uncertainty of explantation; define capsulectomy type; plan pathology/cultures when indicated.	Explantation may be reasonable in selected patients but should not be guaranteed to resolve symptoms. En bloc capsulectomy should be reserved for established implant-associated cancer after appropriate workup [40].
9. Post-intervention follow-up	Measure outcome longitudinally	Baseline symptom score before treatment; follow-up at 3, 6, and 12 months; repeat targeted labs if initially abnormal; document local pathology, culture results, rupture, silicone granuloma, inflammation, or malignancy.	Improvement after explantation supports but does not prove causality. Persistent symptoms require renewed differential diagnosis and rehabilitation-oriented care.

## Data Availability

No new data were created or analyzed in this study. Data sharing is not applicable to this article.

## References

[1] Kaoutzanis C., Winocour J., Unger J., Gabriel A., Maxwell G.P. (2019). The evolution of breast implants. Seminars in Plastic Surgery.

[2] Maxwell G.P., Gabriel A. (2017). Breast implant design. Gland Surg..

[3] Klassen A.F., Pusic A.L., Scott A., Klok J., Cano S.J. (2009). Satisfaction and quality of life in women who undergo breast surgery: A qualitative study. BMC Women’s Health.

[4] Caldarelli M., Rio P., Giambra V., Gasbarrini A., Gambassi G., Cianci R. (2024). ASIA syndrome: State-of-the-art and future perspectives. Vaccines.

[5] e Silva D.N., Gründler C., de Melo Teixeira M.d.G., Horimoto A.M.C., Machado M.A., Frazão I.C., Takita L.C. (2017). Autoimmune syndrome induced by adjuvants (ASIA) after silicone breast augmentation surgery. Plast. Reconstr. Surg. Glob. Open.

[6] Torres-Saavedra F.A., León-Sierra L.P., Rondón-Carvajal J. (2024). ASIA syndrome (autoimmune/inflammatory syndrome induced by adjuvants): Narrative literature review. Rev. Colomb. DE Reumatol..

[7] Shoenfeld Y., Agmon-Levin N. (2011). ‘ASIA’–autoimmune/inflammatory syndrome induced by adjuvants. J. Autoimmun..

[8] Ferreira S., Barros A.S., Marques M. (2025). Breast implant illness: Symptoms, outcomes with explantation and potential etiologies—A systematic review and meta-analysis. Aesthetic Plast. Surg..

[9] Bird G., Niessen F. (2022). The effect of explantation on systemic disease symptoms and quality of life in patients with breast implant illness: A prospective cohort study. Sci. Rep..

[10] Borhani-Khomani K., Kalstrup J., Trøstrup H., Henriksen T.F., Hölmich L.R., Stellander A.K.L. (2024). Self-reported systemic symptoms among women with breast implants. Ugeskr. Laeger.

[11] Bengtson B.P., Van Natta B.W., Murphy D.K., Slicton A., Maxwell G.P., Style 410 U.S. Core Clinical Study Group (2007). Style 410 highly cohesive silicone breast implant core study results at 3 years. Plast. Reconstr. Surg..

[12] Balk E.M., Earley A., Avendano E.A., Raman G. (2016). Long-term health outcomes in women with silicone gel breast implants: A systematic review. Ann. Intern. Med..

[13] Janowsky E.C., Kupper L.L., Hulka B.S. (2000). Meta-analyses of the relation between silicone breast implants and the risk of connective-tissue diseases. N. Engl. J. Med..

[14] Magnusson M.R., Cooter R.D., Rakhorst H., McGuire P.A., Adams W.P., Deva A.K. (2019). Breast implant illness: A way forward. Plast. Reconstr. Surg..

[15] McKernan C.D., Vorstenbosch J., Chu J.J., Nelson J.A. (2022). Breast implant safety: An overview of current regulations and screening guidelines. J. Gen. Intern. Med..

[16] Anderson J.M., Rodriguez A., Chang D.T. (2008). Foreign body reaction to biomaterials. Seminars in Immunology.

[17] Bayston R. (2022). Capsule formation around breast implants. JPRAS Open.

[18] Cronin T.D., Greenberg R.L. (1970). Our experiences with the silastic gel breast prosthesis. Plast. Reconstr. Surg..

[19] Schoberleitner I., Faserl K., Tripp C.H., Pechriggl E.J., Sigl S., Brunner A., Zelger B., Hermann-Kleiter N., Baier L., Steinkellner T. (2024). Silicone implant surface microtopography modulates inflammation and tissue repair in capsular fibrosis. Front. Immunol..

[20] Malik A.F., Hoque R., Ouyang X., Ghani A., Hong E., Khan K., Moore L.B., Ng G., Munro F., Flavell R.A. (2011). Inflammasome components Asc and caspase-1 mediate biomaterial-induced inflammation and foreign body response. Proc. Natl. Acad. Sci. USA.

[21] Swanson K.V., Deng M., Ting J.P.-Y. (2019). The NLRP3 inflammasome: Molecular activation and regulation to therapeutics. Nat. Rev. Immunol..

[22] Dantzer R., O’connor J.C., Freund G.G., Johnson R.W., Kelley K.W. (2008). From inflammation to sickness and depression: When the immune system subjugates the brain. Nat. Rev. Neurosci..

[23] Pajkos A., Deva A.K., Vickery K., Cope C., Chang L., Cossart Y.E. (2003). Detection of subclinical infection in significant breast implant capsules. Plast. Reconstr. Surg..

[24] Tamboto H., Vickery K., Deva A.K. (2010). Subclinical (biofilm) infection causes capsular contracture in a porcine model following augmentation mammaplasty. Plast. Reconstr. Surg..

[25] Alessandri-Bonetti M., Jeong T., Vaienti L., De La Cruz C., Gimbel M.L., Nguyen V.T., Egro F.M. (2023). The role of microorganisms in the development of breast implant-associated anaplastic large cell lymphoma. Pathogens.

[26] Vinci V., Belgiovine C., Janszen G., Agnelli B., Pellegrino L., Calcaterra F., Cancellara A., Ciceri R., Benedetti A., Cardenas C. (2024). Breast implant surface topography triggers a chronic-like inflammatory response. Life Sci. Alliance.

[27] Jaffe E.S., Ashar B.S., Clemens M.W., Feldman A.L., Gaulard P., Miranda R.N., Sohani A.R., Stenzel T., Yoon S.W. (2020). Best practices guideline for the pathologic diagnosis of breast implant–associated anaplastic large-cell lymphoma. J. Clin. Oncol..

[28] Clemens M.W., Myckatyn T.M., Di Napoli A., Feldman A.L., Jaffe E.S., Haymaker C.L., Horwitz S.M., Hunt K.K., Kadin M.E., McCarthy C.M. (2024). American Association of Plastic Surgeons consensus on breast implant–associated anaplastic large-cell lymphoma. Plast. Reconstr. Surg..

[29] Wee C.E., Younis J., Isbester K., Smith A., Wangler B., Sarode A.L., Patil N., Grunzweig K., Boas S., Harvey D.J. (2020). Understanding Breast Implant Illness, Before and After Explantation: A Patient-Reported Outcomes Study. Ann. Plast. Surg..

[30] Moling O., Piccin A., Tauber M., Marinello P., Canova M., Casini M., Negri G., Raffeiner B., Binazzi R., Gandini L. (2016). Intravascular large B-cell lymphoma associated with silicone breast implant, HLA-DRB1* 11: 01, and HLA-DQB1* 03: 01 manifesting as macrophage activation syndrome and with severe neurological symptoms: A case report. J. Med. Case Rep..

[31] Mempin M., Hu H., Chowdhury D., Deva A., Vickery K. (2018). The A, B and C’s of silicone breast implants: Anaplastic large cell lymphoma, biofilm and capsular contracture. Materials.

[32] De Boer M., Colaris M., Van Der Hulst R., Cohen Tervaert J. (2017). Is explantation of silicone breast implants useful in patients with complaints?. Immunol. Res..

[33] Pluvy I., Randrianaridera E., Tahmaz I., Melin M., Gindraux F., Keime C., Ponche A., Petithory T., Pieuchot L., Anselme K. (2025). Breast implant silicone exposure induces immunogenic response and autoimmune markers in human periprosthetic tissue. Biomaterials.

[34] Hallab N.J., Jacobs J.J. (2017). Chemokines associated with pathologic responses to orthopedic implant debris. Front. Endocrinol..

[35] Abdoulkader N., Archibald J., Lignereux M., Lehoux E.A., Catelas I. (2025). NLRP3 inflammasome-dependent and-independent interleukin-1β release by macrophages exposed to wear and corrosion products from CoCrMo implants. PLoS ONE.

[36] Khan I., Minto R.E., Kelley-Patteson C., Singh K., Timsina L., Suh L.J., Rinne E., Van Natta B.W., Neumann C.R., Mohan G. (2023). Biofilm-derived oxylipin 10-HOME–mediated immune response in women with breast implants. J. Clin. Investig..

[37] Jacombs A. (2015). The Consequences and Prevention of Bacterial Biofilm Infection of Silicone Breast Implants. Ph.D. Thesis.

[38] Bassetto F., Scarpa C., Vindigni V., Doria A. (2012). The periprosthetic capsule and connective tissue diseases: A piece in the puzzle of autoimmune/autoinflammatory syndrome induced by adjuvants. Exp. Biol. Med..

[39] Wong D.W., Lam T.K. (2021). Diagnosis, investigation and management of breast implant illness: A narrative review. Australas. J. Plast. Surg..

[40] Tevis S.E., Hunt K.K., Clemens M.W. (2019). Stepwise En Bloc Resection of Breast Implant-Associated Anaplastic Large Cell Lymphoma with Oncologic Considerations. Aesthet. Surg. J. Open Forum.

[41] Thomsen J.L., Christensen L., Nielsen M., Brandt B., Breiting V.B., Felby S., Nielsen E. (1990). Histologic changes and silicone concentrations in human breast tissue surrounding silicone breast prostheses. Plast. Reconstr. Surg..

[42] Kelley N., Jeltema D., Duan Y., He Y. (2019). The NLRP3 inflammasome: An overview of mechanisms of activation and regulation. Int. J. Mol. Sci..

[43] Wurzer P., Hundeshagen G., Cambiaso-Daniel J., Fischer S., Hoflehner H., Spendel S., Lumenta D.B., Kamolz L.P., Kneser U., Hirche C. (2019). Lessons Learned from Breast Implant Registries: A Systematic Review. Ann. Plast. Surg..

[44] Bargon C.A., Becherer B.E., Young-Afat D.A., van Bommel A., Hommes J., Hoornweg M.J., Keuter X., de Fazio S., Melnikov D., Echeverria J.M. (2021). Moving breast implant registries forward: Are they FAIR and Functional?. J. Plast. Reconstr. Aesthet. Surg..

[45] D’orsi G., Giacalone M., Calicchia A., Gagliano E., Vannucchi L., Vanni G., Buonomo O.C., Cervelli V., Longo B. (2024). BIA-ALCL and BIA-SCC: Updates on clinical features and genetic mutations for latest recommendations. Medicina.

[46] Cohen Tervaert J., Mohazab N., Redmond D., Van Eeden C., Osman M. (2022). Breast implant illness: Scientific evidence of its existence. Expert Rev. Clin. Immunol..

